# Impact of type 2 diabetes mellitus on short-term and long-term outcomes of patients with esophageal squamous cell cancer undergoing resection: a propensity score analysis

**DOI:** 10.1186/s40880-018-0275-2

**Published:** 2018-04-27

**Authors:** Wang Yao, Yuqi Meng, Mingjian Lu, Wenzhe Fan, Jinhua Huang, Jiaping Li, Zhihua Zhu

**Affiliations:** 1 Sun Yat-sen University Cancer Center, State Key Laboratory of Oncology in South China, Collaborative Innovation Center for Cancer Medicine, Guangzhou, 510060 China; 2 Department of Interventional Oncology, The First Affiliated Hospital, Sun Yat-sen University, No. 58, Zhongshan Second Road, Guangzhou, 510080 Guangdong P. R. China; 3 Department of Thoracic Surgery, Second Hospital of Lanzhou University, Lanzhou, 730030 Gansu P. R. China; 4 Department of Radiology, Affiliated Cancer Hospital & Institute of Guangzhou Medical University, Guangzhou, 510060 Guangdong P. R. China

**Keywords:** Type 2 diabetes mellitus, Esophageal cancer, Prognosis, Overall survival

## Abstract

**Background:**

The association between type 2 diabetes mellitus (T2DM) and the risk of esophageal cancer remains unclear. The present study aimed to evaluate the impact of T2DM on short-term outcomes and long-term survival in patients with esophageal squamous cell cancer (ESCC).

**Methods:**

The present retrospective study included 862 patients diagnosed with ESCC between January 2001 and December 2010. Among them, 280 patients had T2DM. A 1:1 propensity score-matched cohort consisting of 280 patients with and 280 without T2DM was selected from the 862 patients. The associations between T2DM and clinicopathologic characteristics were assessed using the χ^2^ or Fisher’s exact test. Survival of ESCC patients with and without T2DM was calculated by using the Kaplan–Meier method and compared by using the Cox regression model between the two groups.

**Results:**

The occurrence rate of anastomotic leakage was significantly higher in patients with T2DM than in those without T2DM (*P* < 0.001). In the subgroup with weight loss rate ≤ 5.05%, ESCC patients with T2DM had a significant longer overall survival than did those without T2DM (*P *= 0.003), whereas in the subgroup with weight loss rate > 5.05%, the patients without T2DM showed a longer survival (*P *= 0.001). Univariate and multivariate analysis results showed that T2DM was not an independent prognostic factor for patient survival.

**Conclusions:**

Type 2 diabetes mellitus is not an independent prognostic factor in patients with ESCC. However, the combination of T2DM with severe weight loss would be a predictor of poor prognosis.

## Background

Esophageal cancer is the fifth most common cancer in China, with 287,600 patients newly diagnosed each year, and the fourth most frequent cause of cancer-related deaths, accounting for 210,900 deaths each year [1]. In Asian countries, more than 90% of esophageal cancers are esophageal squamous cell carcinomas (ESCC) [2]. Surgery remains the predominant modality in the management of esophageal cancer [3].

Over the past two decades, the markedly increased morbidity of type 2 diabetes mellitus (T2DM) in most countries has suggested a possible association between diabetes mellitus and cancer [4]. T2DM patients have shown an increased incidence of several cancers, including pancreatic cancer [5], liver cancer [6, 7], colorectal cancer [8], gastric cancer [9], and renal cancer [10]. In contrast, a decreased incidence of prostate cancer has been observed in diabetic patients, suggesting a protective effect of diabetes [11]. However, the effect of T2DM on the prognosis of patients with several types of cancer, including lung cancer, remains unclear [12, 13].

Over the past two decades, the association between T2DM and the risk of esophageal cancer has been examined in different population- and hospital-based settings, but these studies have yielded inconsistent results [14, 15]. A recent meta-analysis reported by Huang et al. [16] has supported the hypothesis that men with diabetes may have a modestly increased risk of esophageal cancer. To date, however, the prognostic significance of T2DM in ESCC has not yet been determined. In the present study, we therefore evaluated the effect of T2DM on the prognosis of ESCC patients who underwent curative surgery.

## Patients and methods

### Study design

The study included patients with histologically proven ESCC who underwent conventional open esophagectomy with 2-field lymphadenectomy at the First Affiliated Hospital of Sun Yat-sen University, the Second Hospital of Lanzhou University, or Sun Yat-sen University Cancer Center between January 2001 and December 2010. Patients were excluded if they (1) had received preoperative or/and postoperative chemotherapy and/or radiotherapy; (2) presented with synchronous primary tumors and/or had previous malignant diseases; (3) underwent R1 or R2 resection; or (4) underwent subtotal/total gastrectomy or had other complications, including hyperthyroidism or active tuberculosis.

A diagnosis of diabetes mellitus is based on a patient’s history of diabetes mellitus and/or the use of antidiabetic mediations in the hospital medical record (all diabetes diagnoses should be made on the basis of classification and diagnosis of Diabetes released in 2015) [17]. Blood sugar levels were rigorously controlled by insulin injections during the perioperative period.

To minimize the effect of potential confounders on selection bias, propensity score-matching analysis was performed. Factors related to combined treatment were entered into the propensity model; these factors included age, sex, smoking status, drinking status, weight loss rate, body mass index (BMI), tumor location, tumor grade, pT category, pN category, pathological tumor/node/metastasis (pTNM) stage, and complications. Patients with and without T2DM were matched one-to-one using the optimal matching method. The adjusted comparisons by propensity scores were based on data from the patients with T2DM. After adjustment for these factors, overall survival (OS) rates, baseline characteristics, and were re-analyzed to explore the prognostic factors for the patients with and without T2DM.

Preoperative evaluation included a complete history, a physical examination, complete blood cell counts, serum biochemistry, chest X-rays, barium swallow test, computed tomography (CT) scans of the chest and abdomen, ultrasonic esophagoscopy, and mandatory cardio-pulmonary assessments.

### Follow-up

Postoperative follow-up and CT scans were performed every 3 months during the first year, every 6 months during the next 2 years, and once every year thereafter. Contrast CT scans of the lower neck, thorax, and upper abdomen, including the liver and adrenal glands, were routinely performed.

Survival status was verified using the best available methods in December 2015, including reviews of outpatient records and direct telecommunication with each patient or his/her family. T2DM history, weight loss, BMI, smoking status, and drinking status were determined by reviewing each patient’s medical records at the first visit to a hospital. Weight loss rate was defined as the following formula [18]:

$$ {\text{Weight loss rate }} = \frac{{{\text{weight1}} - {\text{weight2}}}}{\text{weight1}} $$

weight1: body weight measured on the first day of admission.

weight2: body weight measured before leaving the hospital.

Because few reports have defined weight loss rate, this parameter was empirically divided into four categories: 0, > 0 to ≤ 5%, > 5% to ≤ 10%, and > 10%.

Complications were estimated from clinical, laboratory, and imaging data. The pTNM stage of each patient was defined according to the criteria of the American Joint Committee on Cancer staging system (2010).

### Statistical analysis

All statistical analyses were performed using the SPSS 19.0 software package (IBM, Chicago, IL, USA). OS was defined as the time from the date of surgery to the date of death or final clinical follow-up. All patients lost to follow-up or being alive at the last follow-up were censored. Associations between T2DM and clinicopathologic characteristics were assessed using the χ^2^ or Fisher’s exact test. Survival rates were calculated with Kaplan–Meier curves and compared with log-rank tests. Univariate and multivariate cox regression analyses were controlled for the variables mentioned above. A two-sided *P* value < 0.05 was considered statistically significant. Receiver operating characteristic (ROC) curve was constructed using Medcalc 12.0 (Medcalc Company, Brussels, Belgium). Propensity score-matched analysis was performed using R software (TIBCO, Silicon Valley, CA, USA).

## Results

### Patient characteristics

**Fig. 1:**
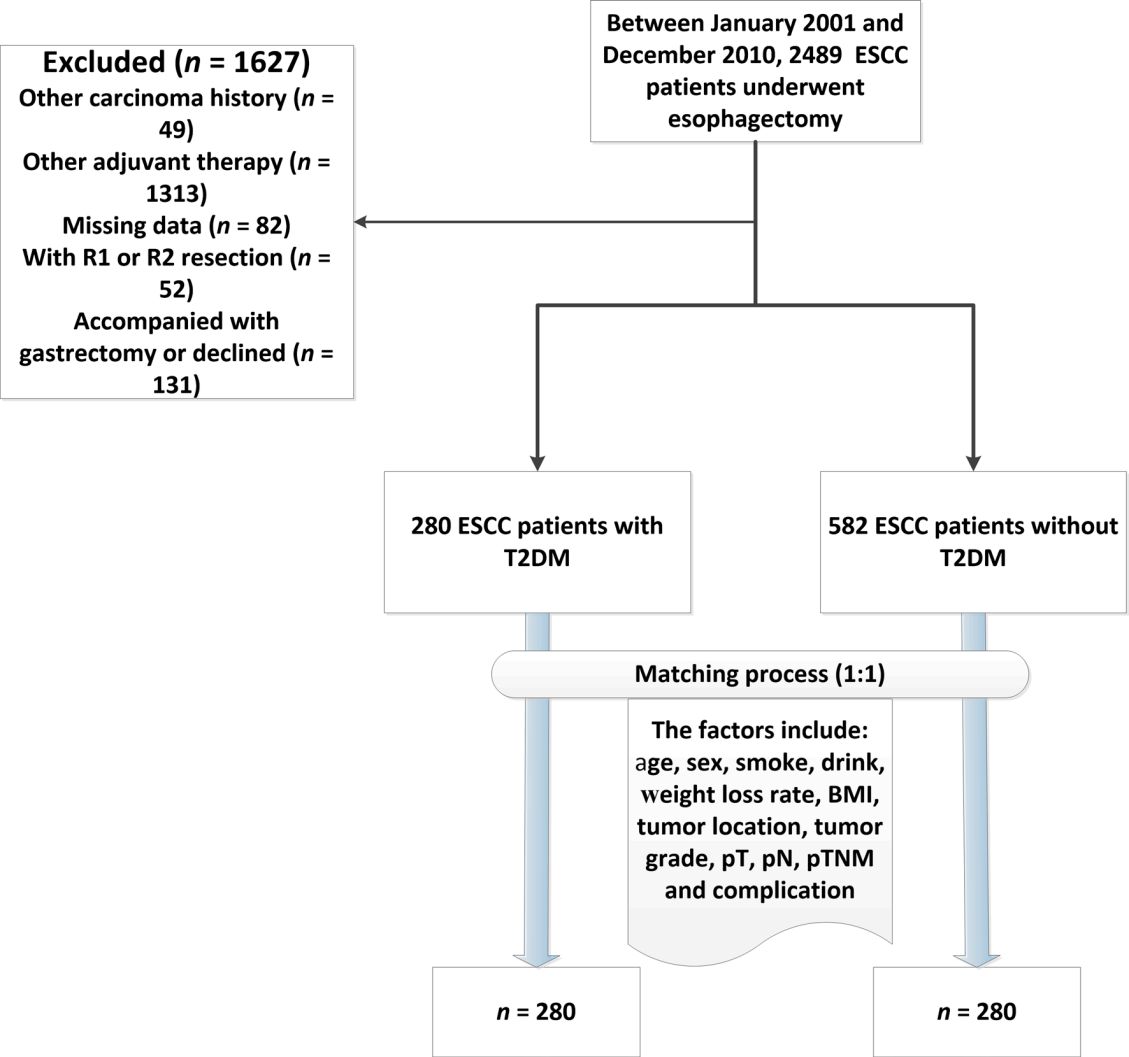
The flow diagram of patient selection. *ESCC* esophageal squamous cell cancer, *T2DM* type 2 diabetes mellitus, *pT* pathological tumor, *pN* pathological node, *pTNM* pathological tumor/node/metastasis, *BMI* body mass index

Between January 2001 and December 2010, a total of 2489 patients with ESCC underwent open esophagectomy at the three institutions. The study cohort consisted of 862 patients with ESCC who underwent surgery as primary therapy; 638 (74.0%) of them were male (Fig. 1). Before propensity score matching, the mean age of all patients was 60.1 ± 9.2 years. The median weight loss rate and BMI of these patients were 0.0% (range, 0–18.8%) and 22.06 kg/m^2^ (range, 14.98–32.96 kg/m^2^), respectively. After propensity score matching, mean age was 61.8 ± 9.0 years, the median weight loss rate was 3.1% (range, 0–18.8%), and the median BMI was 22.75 kg/m^2^ (range, 14.98–32.96 kg/m^2^). The characteristics of the 280 matched T2DM and non-T2DM patients are shown in Table 1. All further statistical analyses were performed on this population.

**Table 1:** Characteristics of 862 ESCC patients with or without T2DM

Characteristic	Before matching				After matching			
	No. of cases (%)	With T2DM (%)	Without T2DM (%)	*P* value^a^	No. of cases (%)	With T2DM (%)	Without T2DM (%)	*P* value^a^
Age (years)				0.012				0.499
≤ 45	48 (5.6)	4 (1.4)	44 (7.6)		13 (2.3)	4 (1.4)	9 (3.2)	
46–59	370 (42.9)	112 (40.0)	258 (44.3)		225 (40.2)	112 (40.0)	113 (40.4)	
≥ 60	444 (51.5)	164 (58.6)	280 (48.1)		322 (57.5)	164 (58.6)	158 (56.4)	
Sex				0.024				0.330
Female	224 (26.0)	92 (32.9)	132 (22.7)		195 (34.8)	92 (32.9)	103 (36.8)	
Male	638 (74.0)	188 (67.1)	450 (77.3)		365 (65.2)	188 (67.1)	177 (63.2)	
Smoking status				< 0.001				0.798
Yes	362 (42.0)	160 (57.1)	202 (34.7)		317 (56.6)	160 (57.1)	157 (56.1)	
No	500 (58.0)	120 (42.9)	380 (65.3)		243 (43.4)	120 (42.9)	123 (43.9)	
Drinking status				0.060				0.754
Yes	638 (74.0)	224 (80.0)	414 (71.1)		445 (79.5)	224 (80.0)	221 (78.9)	
No	224 (26.0)	56 (20.0)	168 (28.9)		115 (20.5)	56 (20.0)	59 (21.1)	
Weight loss rate				0.705				0.976
0	474 (55.0)	144 (51.4)	330 (56.7)		292 (52.1)	144 (51.4)	148 (52.9)	
> 0% to ≤ 5%	152 (17.6)	56 (20.0)	96 (16.5)		102 (36.4)	56 (20.0)	46 (16.4)	
> 5% to ≤ 10%	182 (21.1)	60 (21.4)	122 (21.0)		126 (22.5)	60 (21.4)	66 (23.6)	
> 10%	54 (6.3)	20 (7.2)	34 (5.8)		40 (7.1)	20 (7.1)	20 (7.1)	
BMI (kg/m^2^)				0.006				0.409
< 18.5	76 (8.8)	8 (2.9)	68 (11.7)		34 (6.0)	8 (2.9)	26 (9.3)	
≥ 18.5 to < 25.0	622 (72.2)	208 (74.3)	414 (71.1)		393 (70.2)	208 (74.3)	185 (66.1)	
≥ 25.0	164 (19.0)	64 (22.8)	100 (17.2)		133 (23.8)	64 (22.9)	69 (24.6)	
Tumor location				0.052				0.590
Upper	58 (6.7)	20 (7.1)	38 (6.5)		34 (6.0)	20 (7.1)	14 (5.0)	
Middle	560 (65.0)	160 (57.2)	400 (68.7)		337 (60.2)	160 (57.1)	177 (63.2)	
Lower	244 (28.3)	100 (35.7)	144 (24.8)		189 (33.8)	100 (35.7)	89 (31.8)	
Tumor grade				0.206				0.992
G1	232 (26.9)	80 (28.6)	152 (26.1)		148 (26.4)	80 (28.6)	68 (24.3)	
G2	386 (44.8)	120 (42.8)	266 (45.7)		264 (47.2)	120 (42.9)	144 (51.4)	
G3	244 (28.3)	80 (28.6)	164 (28.2)		148 (26.4)	80 (28.5)	68 (24.3)	
pT category				0.231				0.236
T1	48 (5.6)	8 (2.9)	40 (6.9)		18 (3.2)	8 (2.9)	10 (3.6)	
T2	232 (26.9)	76 (27.1)	156 (26.8)		163 (29.1)	76 (27.1)	87 (31.1)	
T3	582 (67.5)	196 (70.0)	386 (66.3)		379 (67.7)	196 (70.0)	183 (65.3)	
pN category				0.284				0.870
N0	476 (55.2)	144 (51.4)	332 (57.1)		285 (50.9)	144 (51.4)	141 (50.4)	
N1	242 (28.1)	80 (28.6)	162 (27.8)		172 (30.7)	80 (28.6)	92 (32.8)	
N2	124 (14.4)	52 (18.6)	72 (12.4)		96 (17.1)	52 (18.6)	44 (15.7)	
N3	20 (2.3)	4 (1.4)	16 (2.7)		7 (1.3)	4 (1.4)	3 (1.1)	
pTNM stage				0.161				0.947
Stage I	46 (5.3)	8 (2.9)	38 (6.5)		20 (3.6)	8 (2.9)	12 (4.3)	
Stage II	450 (52.2)	140 (50.0)	310 (53.3)		273 (48.7)	140 (50.0)	133 (47.5)	
Stage III	366 (42.5)	132 (47.1)	234 (40.2)		267 (47.7)	132 (47.1)	135 (48.2)	
Complication								
None	718 (83.3)	216 (77.1)	502 (86.3)	0.018	458 (81.8)	216 (77.1)	242 (86.4)	0.004
Anastomotic leakage	82 (9.5)	48 (17.1)	34 (5.8)	0.001	60 (10.7)	48 (17.1)	12 (4.3)	< 0.0001
Pneumonia	32 (3.7)	8 (2.9)	24 (4.1)	0.515	22 (3.9)	8 (2.9)	14 (5.0)	0.192
Others^b^	30 (3.5)	8 (2.9)	22 (3.8)	0.624	20 (3.6)	8 (2.9)	12 (4.3)	0.363
Total	862	280	582		560	280	280	

*ESCC* esophageal squamous cell carcinoma, *T2DM* type 2 diabetes mellitus; *Non-DM* patients without type 2 diabetes mellitus; *BMI* body mass index, *pT* pathological tumor, *pN* pathological node, *pTNM stage* pathological tumor/node/metastasis

^a^ Chi square test or Fisher’s exact test

^b^ Other complications include surgical wound infection, chyle leakage, cardiac complications, and cerebral infarction

### Patient characteristics by T2DM

Of the 862 patients, 280 (32.5%) had T2DM and 582 (67.5%) did not. These two groups were well matched in weight loss rate, tumor location, tumor grade, pT category, pN category, and pTNM stage. However, the patients with T2DM were significantly older (*P* = 0.012), had higher percentage of smoking (*P* < 0.001), had a significantly higher percentage of female (*P* = 0.024), had a significantly higher BMI (*P* = 0.006), had significantly higher probability of no complications (*P* = 0.018), and had higher rates of postoperative anastomotic leakage (*P* = 0.001) than the patients without T2DM. After propensity score matching, all characteristics in the two groups were well matched, expect for the probability of no complication (*P* = 0.004) and postoperative anastomotic leak (*P* < 0.001) (Table 1).

### Overall survival and T2DM

**Fig. 2:**
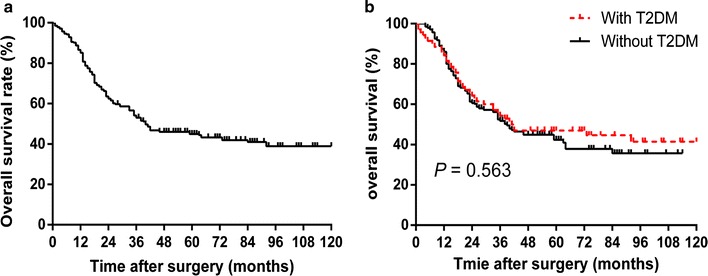
The Kaplan–Meier overall survival curves for **a** the entire cohort of patients with esophageal squamous cell cancer and **b** esophageal cancer patients with and without type 2 diabetes mellitus (T2DM)

The median and mean OS were 45.5 and 39.0 months, respectively. The 1-, 3-, and 5-year OS rates for the entire patient cohort were 86.6%, 53.2%, and 30.2%, respectively (Fig. 2a). Log-rank test showed that the OS rate tended to be higher in ESCC patients with T2DM than in those without T2DM, but the difference between them was not significant (*P *= 0.563, Fig. 2b).

**Fig. 3:**
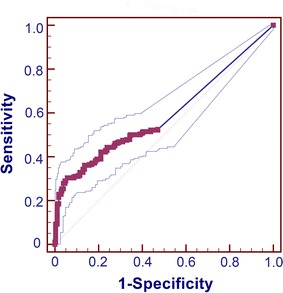
The area under receiver operating characteristic (ROC) curves for weight loss rate in patients with esophageal squamous cell cancer

The modeled area under curve (AUC) for weight loss rate was 0.593 (*P* < 0.001, 95% confidence interval [CI] 0.551–0.634; Fig. 3). The diagnostic value of the weight loss rate was significantly associated with AUC, with the cutoff points for weight loss rate being 5.05%; the sensitivity and specificity of cutoff points were 0.616 and 0.595, respectively.

**Fig. 4:**
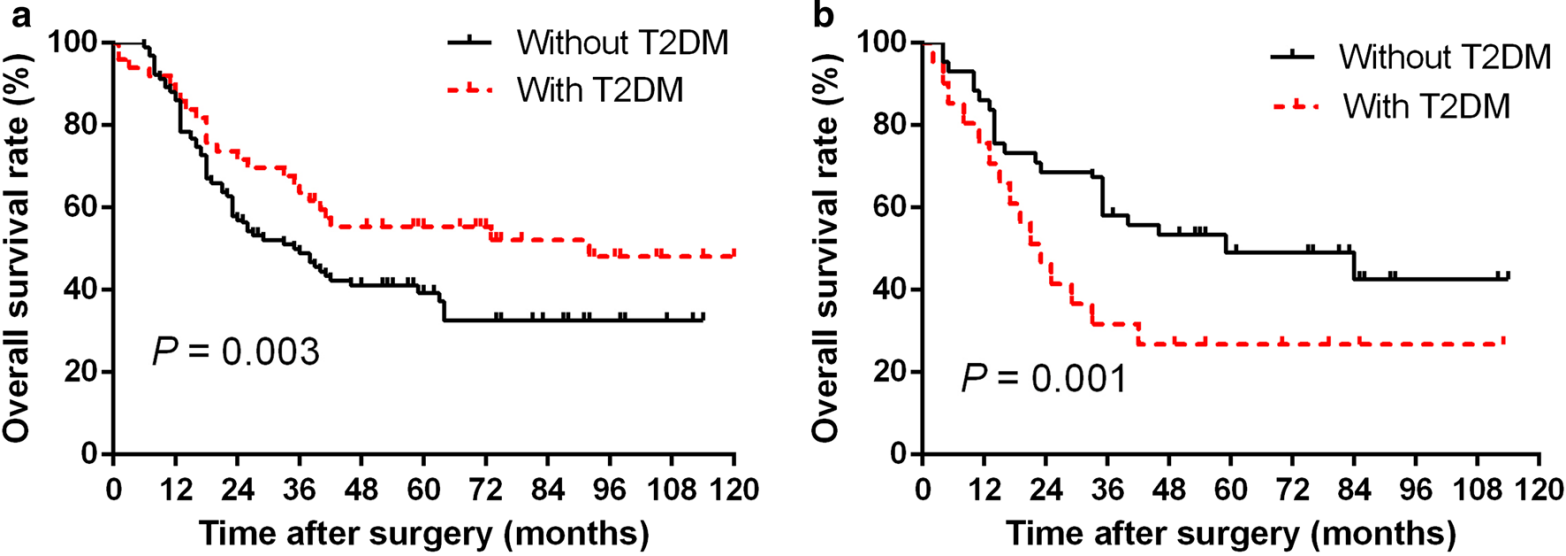
Overall survival curves for the esophageal cancer patients with weight loss rate ≤ 5.05% (**a**) and > 5.05% (**b**), retrospectively, according to the presence or absence of type 2 diabetes mellitus

Subgroup analysis of patients with low weight loss rate (≤ 5.05%) showed that survival was significantly longer in patients with T2DM than in those without T2DM (*P *= 0.003, Fig. 4a). In contrast, analysis of patients with high weight loss rate (> 5.05%) showed that survival was significantly shorter in patient with T2DM than in those without T2DM (*P *= 0.001, Fig. 4b).

### Univariate and multivariable analyses

Univariate analysis showed that T2DM was not significantly associated with patient survival. In contrast, sex (hazard ratio [HR] 0.769; *P* = 0.030), weight loss rate (HR 1.168; *P* = 0.005), tumor grade (HR 1.489; *P* < 0.001), pT category (HR 1.642; *P* < 0.001), pN category (HR 1.820; *P *< 0.001), and pTNM stage (HR 2.270; *P* < 0.001) were significantly associated with the survival of patients with ESCC (Table 2). In multivariate analysis, only tumor grade (HR 1.373; *P *< 0.001) and pN category (HR 1.474; *P *= 0.001) were significantly associated with patient survival (Table 2).

**Table 2:** Univariate and multivariate cox regression analysis for overall survival in patients with ESCC

Characteristic	Univariate analysis			Multivariate analysis		
	HR	95% CI	*P* value	HR	95% CI	*P* value
Age (years)						
≤ 45 vs. 46–59 vs. ≥ 60	1.002	0.990–1.015	0.720	–	–	–
Sex						
Female vs. male	0.769	0.607–0.975	0.030	0.865	0.680–1.101	0.239
Smoking status						
Yes vs. no	1.176	0.944–1.465	0.149	–	–	–
Drinking status						
Yes vs. no	1.195	0.922–1.548	0.178	–	–	–
Weight loss rate						
0 vs. > 0% to ≤ 5.0% vs. > 5.0% to ≤ 10.0% vs. > 10.0%	1.168	1.047–1.302	0.005	1.118	1.000–1.251	0.060
BMI (kg/m^2^)						
< 18.5 vs. ≥ 18.5 to < 25.0 vs. ≥ 25.0	0.970	0.779–1.207	0.784	–	–	–
Tumor location						
Upper vs. middle vs. lower	0.932	0.767–1.134	0.483	–	–	–
Tumor grade						
G1 vs. G2 vs. G3	1.489	1.271–1.745	< 0.001	1.373	1.164–1.621	< 0.001
pT category						
T1 vs. T2 vs. T3 vs. T4	1.642	1.308–2.062	< 0.001	1.257	0.982–1.611	0.070
pN category						
N0 vs. N1 vs. N2 vs. N3	1.820	1.594–2.079	< 0.001	1.474	1.181–1.839	0.001
pTNM stage						
Stage I vs. stage II vs. stage III	2.270	1.832–2.813	< 0.001	1.237	0.872–1.754	0.233
Complications						
None vs. anastomotic leakage vs. pneumonia vs. others	1.118	0.961–1.299	0.148	–	–	–
T2DM						
With T2DM vs. without T2DM	0.896	0.719–1.116	0.326	–	–	–

*ESCC* esophageal squamous cell carcinoma, *BMI* body mass index, *T2DM* type 2 diabetes mellitus, *pT* pathological tumor, *pN* pathological node, *pTNM stage* pathological tumor/node/metastasis, *HR* hazard ratio, *95% CI* 95% confidence interval, *–* no comparison

## Discussion

The present study found no significant difference in OS between the ESCC patients with T2DM and those without T2DM. Analysis of the subgroup of patients with weight loss rate ≤ 5.05% showed that OS was significantly longer in patients with T2DM than in those without T2DM, whereas analysis of the subgroup with weight loss rate > 5.05% showed that OS was significantly shorter in patients with T2DM than in those without T2DM.

Although the underlying impact of T2DM on ESCC prognosis is unclear, several possible mechanisms have been proposed. Metabolic abnormalities associated with diabetes, such as insulin resistance, compensatory hyperinsulinemia, and elevated levels of bioactive insulin-like growth factor and/or chronic inflammation, can stimulate cancer cell mitogenesis, proliferation, invasion, and metastasis, enhancing tumorigenesis and progression [19]. Hyperinsulinemia has been shown to increase bioavailable estrogen and testosterone [20], sex hormones that play a role in the development of ESCC [21]. In addition, early evidence suggests that some treatments of diabetes may affect cancer risk [19]. For example, exposure to metformin was reported to be significantly associated with prolonged survival in patients with various types of cancer, including thyroid cancer [22], colorectal cancer [23], laryngeal squamous cell cancer [24], melanoma [25], and pancreatic cancer [26]. Delayed gastric emptying is frequent in patients with diabetes, increasing the likelihood of gastric/esophageal reflux, a risk factor for ESCC [27–29]. However, the association between T2DM and ESCC remains complicated and ambiguous, including a need for additional studies.

The present study showed that T2DM was associated with several clinical factors of patients with ESCC. In accordance with the previous studies [30, 31], we found that T2DM was more likely to occur in older patients, women, patients with a history of smoking, and patients with a high BMI. Our findings also demonstrated that the rate of anastomotic leakage was almost threefold higher in patients with T2DM than in those without T2DM. One of the adverse consequences of hyperglycemia is wound healing delay, which may lead to anastomotic leakage [31].

Interestingly, survival benefits seen in patients with T2DM have been reported to depend on more frequent and regular consultations, resulting in an earlier diagnosis [11]. However, because of the drawbacks of our clinical follow-up data, we could not compare the frequencies of consultations between the two groups of patients.

Despite the potential contributions of T2DM to survival benefits in patients with ESCC, the effect of T2DM on metabolism cannot be ignored. Nutritional support remains a cornerstone in the management of ESCC and has been shown to ameliorate patients’ tolerance of treatment, quality of life, and long-term outcomes. The combination of ESCC and T2DM may present great challenges to patients in a multitude of nutrition-related areas. Severe weight loss, which may result from dysphagia, cancer-related cachexia, and/or uncontrolled diabetes mellitus, is a prognostic factor in patients with ESCC [32]. Based on these considerations, a logical and plausible interpretation of our findings was that severe weight loss may mask the negative prognostic effect of T2DM in patients with ESCC.

Despite showing the impact of T2DM on the prognosis of ESCC patients, the present study had several limitations. First, our study was a retrospective study, and the number of patients was relatively small, particularly in subgroup analyses, which increases the likelihood of spurious associations. Second, this study did not evaluate the usage of anti-diabetic drugs and the frequency of consultations because of incomplete clinical data. Therefore, additional investigations, especially multi-center prospective randomized controlled trials with adequate statistical power are required to determine whether and how T2DM affects the survival of patients with ESCC.

## Conclusions

Type 2 diabetes mellitus was not found to be an independent prognostic factor of OS in patients with ESCC. However, the combination of T2DM and severe weight loss may be used as a predictor of poor prognosis.

## Abbreviations

BMI: body mass index
EC: esophageal cancer
ESCC: esophageal squamous cell cancer
HR: hazard ratio
T2DM: type 2 diabetes mellitus
OS: overall survival
pT: pathological tumor
pN: pathological node
pTNM: pathological tumor/node/metastasis
95% CI: 95% confidence interval
CT: computed tomography
ROC: receiver operating characteristic
